# Using Social Media Data to Evaluate Urban Parks Use during the COVID-19 Pandemic

**DOI:** 10.3390/ijerph182010860

**Published:** 2021-10-15

**Authors:** Mihai Răzvan Niță, Miruna Arsene, Giorgiana Barbu, Alina Gabriela Cus, Mihail Ene, Ramona Mihaela Serban, Constantin Marian Stama, Larissa Nicoleta Stoia

**Affiliations:** 1 Centre for Environmental Research and Impact Studies, University of Bucharest, 010041 Bucharest, Romania; mihairazvan.nita@g.unibuc.ro; 2 Faculty of Geography, University of Bucharest, 010041 Bucharest, Romania; miruna.arsene@s.unibuc.ro (M.A.); giorgianabarbu22@gmail.com (G.B.); alina.cus@s.unibuc.ro (A.G.C.); mihail.ene@s.unibuc.ro (M.E.); ramona.serban@s.unibuc.ro (R.M.S.); constantin.stama@s.unibuc.ro (C.M.S.)

**Keywords:** social media, urban parks, visitors, pandemic period, urban planning

## Abstract

In the context of increasing urbanization and associated economic, social and environmental challenges, cities have increasingly acknowledged the importance of urban parks in delivering social, economic and environmental benefits to the population. The importance has been demonstrated also during the COVID-19 pandemic that generated lockdowns and reduced the capacity of urban inhabitants in accessing such benefits. The present study aims to determine how the presence in urban parks was reflected on social media during the pandemic period of 2020. We examined Instagram posts associated with a sample of eight urban parks in Bucharest, Romania and also the entire history of Google reviews between January and August 2020. The selection of parks was made according to their size, location in Bucharest, previous reported number of visitors and profile of attractiveness. Results revealed that the peak period of the COVID-19 pandemic and the first initiation of the lockdowns strongly affected the recreation and leisure activities that people performed almost daily in the parks of Bucharest. Reviews and comments of the population were not that focused on the pandemic even after the restrictions were lifted, but they evidenced the positive and negative aspects of each park. Our results can represent a useful instrument for local administrations in determining both the flow of visitors but also their perceptions towards the endowments, landscape and most important management of urban parks.

## 1. Introduction

As a response to increasing challenges of urbanization and changing weather and climate conditions, urban green infrastructure (UGI) has emerged as a concept to increase resilience within urban boundaries [1]. Green spaces, especially parks, are important determinants of the quality of life in cities [2] and are defined as large green areas including various characteristics such as trees, playgrounds and other smaller green areas [3]. Incorporating urban green infrastructure features in the urban landscapes brings a series of benefits [4], which include those related to human health [5], social interactions and inclusion, urban and rural connectivity, work opportunities, climate change management [6].

For optimal planning and management of urban parks, an important step is to quantify users’ visits and to understand the driving factors of their visits [7]. Traditional methods of quantifying the visitor numbers include surveys and on-site observations, but they have been found to be both time and labor consuming while also being costly and easily subjected to bias [8,9]. A more modern approach to assessing the number of visits to urban parks and the factors that influence such visits consist of analyzing location-based social media data sources, which have been proven to be resourceful as management tools, specifically for crowd control and in the process of allocating recreational resources [10].

The understanding of factors that influence park use is an ever-emerging research focus, with most studies demonstrating that visitation rates are positively influenced by recreational features such as trails, sports and pet-friendly facilities [8,9], socializing opportunities for friends, families and different ethnic groups [11], aesthetic values of landscapes [12], cultural heritage [13], inspirational, spiritual, religious and existence values [14].

Although they make a major contribution to the day-to-day activities, most parks do not serve everyone in the community equally [15]. Visitor typologies are differentiated by age, gender, education, lifestyle, health or mobility capacity [16]. People use parks for different activities, both personal and professional. The most used activity is recreation, leisure. Over time it has been observed that older people use parks to exercise, improve their health or to decrease the feeling of loneliness [17]. Children and young people use parks from two perspectives: passive (socializing) and active by carrying out sports activities, walking and involvement in various outdoor games [18]. Although young people represent the largest number of park users, their time outdoors has decreased significantly in recent years due to limited opportunities and lack of experience in nature [11].

The present COVID-19 pandemic represents an unseen before threat to the global human health, economy and societies [19]. With social distancing regarded as one of the main effective approaches to halt the spreading of the virus [20], the implementation of such measure on a worldwide scale has caused abrupt and profound disruptions of the everyday lives of millions of people, with a likely impact on the well-being of populations that reside in high-density urban environments [21]. Since the beginning of the epidemic, citizens’ demands for open green spaces and parks has increased significantly, highlighting their role and major importance in providing cultural and health benefits [22].

Recent research has shown that during the pandemic, people suffered behavioral changes [23], with an increasingly reported attitude behavior nexus in leisure and travel [24] and increasingly turned their attention to parks near the home to enjoy clean and fresh air, exercise and meditation [25]. Nevertheless, in Latin America, strict restrictions to park use have been proven to increase the pre-existing intra-urban health inequalities [26]. Travel limitation, as necessary as it is to diminish the infection rates, increases stress among communities and complicates the role of parks in assuring psychological and physiological capacity to cope with a crisis [27].

According to the data provided by the Our World Data platform, there were major changes in the number of park visitors throughout the pandemic in Romania. Data show that during the lockdown in March–May 2020, the number of visitors to parks decreased considerably. Bucharest, as of November 2020, has registered a 35% decrease in park visitation compared to the baseline of the Google Mobility Report, while in Romania overall the decrease is situated at 36% [28]. While these numbers provide a cause of concern for the well-being of Romanian citizens, researchers [29] propose that the reports are less valid for outdoor recreation and park visitation than other types of mobility activities which are not affected by the seasonal changes. For park mobility, a more representative baseline and the weather factor should be taken into consideration to increase the data validity.

Google Mobility Reports are sets of data based on location information from Google Users with purpose in showing how communities are moving around differently due to COVID-19 [28]. For parks, the reports provide mobility information for the following categories: national parks, public beaches, marinas, dog parks, plazas and public gardens. Exposure in nature or green space, as well as carrying out physical activities on a regular basis, for both physical and mental health, is a beneficial element in protecting the body and limiting the damage caused by the COVID-19 virus [30].

The attractiveness of green areas such as urban parks is often associated with residents’ preferences and expectations regarding among others the general landscaping of the park, its endowments or facilities for different recreation or leisure activities [31]. The most important thing to know about green spaces’ attractiveness is that it can be induced by the history of the location, the use of infrastructure, proximity, location or complexity of the infrastructure. On the other hand, attractiveness can be correlated with the physical characteristics of a green space, taking into account also the surroundings’ context [32]. Sugiyama [33] suggested that the attractiveness of a green space and the activity options offered by that space may be more relevant to physical activity than the number of open spaces available.

The methods for classifying and quantifying the benefits offered by urban parks are either qualitative (for the evaluation of recreational spaces and social benefits) or quantitative (ecological or economic benefits). An ecological benefit is improving urban air quality, conserving biodiversity by creating support habitats for local flora and fauna species. For social benefits, improving the aesthetics of the urban landscape, space for recreation, creating opportunities for socialization, spaces for playing sports and improving the health of the population. For economic benefits, increasing the property value of nearby properties, reducing energy consumption by maintaining constant local temperatures or direct incomes from activities in the park.

Social networks offer many ways and opportunities to understand human–nature interactions. Photographs posted by people on social networks are useful indicators in the analysis of the ways of recreation in various places such as green spaces and parks [34]. Currently, the analysis of the frequency of visits to parks via social networks can be useful for the purposes of managing the flow of visitors and has high potential to structure the information based on the data obtained [10]. In general, photos and reviews found online contain various information about the user profile, reflect the activities that people carry out in the park, their interests and aesthetic values, as well as information about their sentimental and emotional status.

According to recent studies, the way of collecting data using social media platforms is considered a more simplified version that can obtain in an easy manner a large volume of data, although its precision is highly dependent on a sound methodological approach in the study design. Comparing the use of social media in data collection and analysis with classical methods such as questionnaire or observation sheets, it appears that the use of traditional methods requires a considerable amount of work [35]. Most of the time, the information received from the analyzed persons has questionable accuracy, often due to objective behavior that can deviate from a subjective description [36]. The use of innovative methods of research is gaining traction, especially as user-generated content can provide a valuable tool for easy data collection [37] and methods such as content analysis provide in-depth detailed results [38].

The main aim of our study is to evaluate how the use of urban parks changed due to the COVID-19 pandemic and how it was reflected on social media. To fulfill the purpose of this study, we identified the main characteristics of eight selected case study parks in Bucharest (Romania), how they were used during the COVID-19 pandemic, as well as the typologies of activities carried out and we described them in the following paragraphs. The main objectives underlying this study are as follows: (i) to analyze the online presence of inhabitants in the eight selected urban parks; (ii) to evaluate the opinions and the main activities that visitors post about; (iii) to analyze the involvement of public and its general position on the subject as expressed in their posts and photos.

## 2. Materials and Methods

### 2.1. Study Area

Bucharest is the main urban center of Romania [39] being located in the Plain of Vlasia, subdivision of the Romanian Plain at an average altitude of about 90 m [40]. Bucharest is an interesting case in terms of spatial and demographic changes that existed over time [41], thus after the fall of the communist regime, the city is experiencing a chaotic development, particularly directed towards the urban planning of residential assemblies, without including new green spaces [42]. Once considered the “city of gardens”, today Bucharest is covered by numerous urban problems that affect both the quality of the environment, as well as the well-being and quality of life of the inhabitants [43]. Today, the city of Bucharest has about 103 parks included in various site categories, such as metropolitan, municipal, neighborhood and transit parks; many of them have a great historical significance and a special landscape value. In Bucharest, parks are the main provider of leisure and regulatory services between natural spaces and those built [44]. For our analysis, we chose eight parks included in various site categories (Table 1), such as Cismigiu Park, Tineretului, Circului, Drumul Taberei, Tei, Sebastian, Izvor and Pacii. The parks range from 820 (Izvor) to 7800 (Tineretului) average number of visitors per weekend day—obtained from field observations [44]. The accessibility of visitors indicates the position of the park in the city, relations with neighboring proximities and residential areas, as well as the connection with public transportation, and was derived from spatial analysis into: parks with high accessibility assured by public transport, monopolistic position at neighborhood level, low accessibility [44].

Cismigiu Park it is one of the oldest parks in Bucharest, dating back to 1854 [45]. This Park falls into the category of metropolitan parks and is located in the central area of the city, which makes it very accessible to the population because it is served by a well-developed transport network. Tineretului Park is an important component of the green space system in Bucharest [46] located in the south of the city, being constituted as green space in 1973 and currently having an area of about 102 ha. Today, Tineretului Park is hosting numerous activities for children, playgrounds, but also considered an important green area in terms of relaxation activities, cycling or socializing.

Circului Park is a green space included in the municipal category of parks in Bucharest, having an area of 17.2 ha. The attractiveness of the park is given especially by the presence of a central lake, which is covered almost entirely by water lilies, an area with great natural landscape value. Drumul Taberei Park with an area of 11.8 ha is an important natural element within the urban landscape. [47].

Tei Park is located in the northern part of Bucharest. With an area of 8.5 ha, it is one of the main green areas of sector 2, next to the Circului Park. Recently, the park has been re-designed with facilities similar to amusement parks and other attractions, which has led to an increase in the attractivity for children. Sebastian Park is a neighborhood park with an area of 2.5 ha located in the south-west area of the city. The park is most frequented by people who live nearby, especially the elderly or people who want to relax, to meditate.

**Figure 1:**
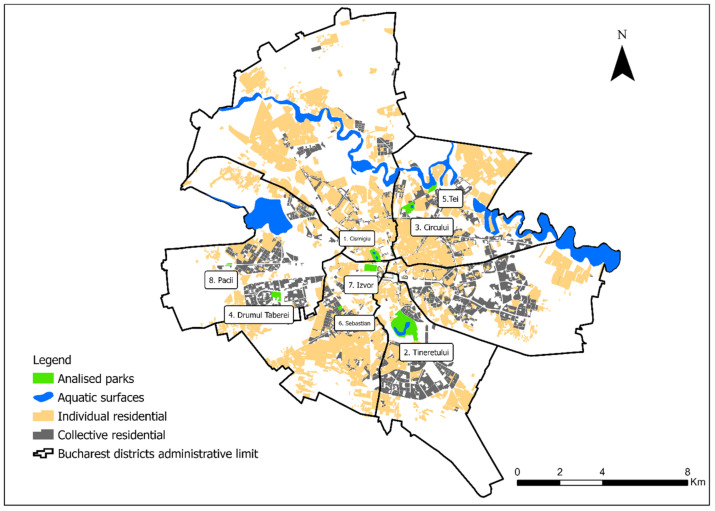
Distribution of the analyzed parks in Bucharest (source: own elaboration).

Păcii Park was founded during the communist period and has an area of 1 ha. It is a neighborhood park located in the 6th sector of Bucharest and represents the main green space in its western extremity (Figure 1). Izvor Park is located in the central area of Bucharest, being one of the parks developed after 1976 [48]. It has an area of 15.9 ha and is included in the category of transit parks. Izvor Park has a low attractiveness especially because of its proximity to Cismigiu Park which is a park of metropolitan importance.

### 2.2. Study Design

We examined Google Maps (web mapping platform offered by Google, Google LLC, Mountain View, CA, USA) for reviews of Bucharest’s parks and Instagram (Facebook Company, Menlo Park, CA, USA) for posts which had the selected parks as the tagged location. Such a method of collecting user-generated content for scientific research has been increasingly been used with large volumes of data available in this method [37]. We distributed the parks randomly among three teams comprised of two Romanian native and English proficient coders, for each selected platform. We selected all Google Maps reviews that included text aside from stars rating, from the period of January–August 2020 and all Instagram posts for the same period (Table 2).

Databases were built using Microsoft Excel Spreadsheet Software (Microsoft Corporation, Redmond, WA, USA) for the data extracted from Google and Instagram, with extracted information regarding data relevant for conducting the relevant analysis for this study’s objectives.

**Figure 2:**
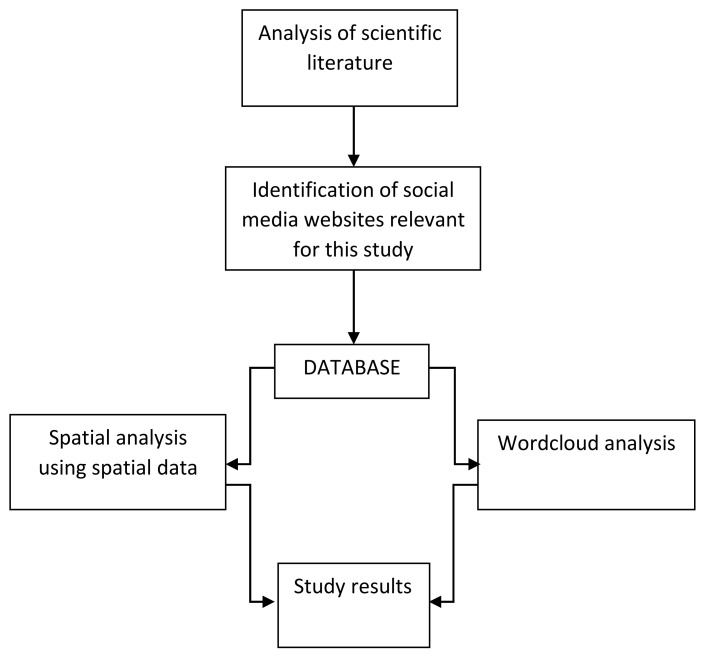
Schematic study design.

The database (Figure 2) included common information for both websites such as the time, the language of the posts and mentions of the pandemic, while also referring to review-specific data for Google and post/pictures-specific data for Instagram. For the word cloud analysis, we used the qualitative data from the Google database, which included the visitors’ reviews of the parks. For similarity of the data, all the initial Romanian reviews were translated into English. Content analysis is increasingly being used as a method of extracting relevant results from large volumes of data [38].

For Google Reviews we searched for information regarding the aspects addressed in the review (either as a general remark, or they are focused on specific elements such as recreation, social relations, aesthetic values, cultural aspects, inspirational and spiritual values, existence values), as well as extracting the exact body of text to search for specific aspects and those related to the COVID-19 pandemic. Using data derived from mobile phones of users has been increasingly been used for tracking down the effects of the COVID-19 pandemic on the behavior of visitors [49]. For Instagram, we added information about the type of post and pictures using a coding system (selfie, personal activities, buildings, animals, plants, general landscapes), similar to other studies who used social media data to understand urban green space [50].

In order to analyze the evolution of social media posts for the studied period, we aggregated data from the databases into a table with the monthly number of posts from both platforms, for each park. Then, for a better visualization of the phenomenon, we built charts and selected those which showed the highest variations in the number of posts. Using ArcInfo (ESRI, Redlands, CA, USA). we represented some of the parameters analyzed on maps of Bucharest.

## 3. Results

The low number of posts in March, April and May (Table 3) coincides with the lockdown period of the pandemic, in which the parks were closed to the population. Before the lockdown period, Tineretului Park had the most visitors, if we look at the number of posts between January and early March. Cismigiu Park is a special element, in which it resulted from the evolution of the posts that it did not have a high number of visitors, a cause being its location in the city center.

Although, in general, Cismigiu Park experiences a high number of visitors, especially on weekends, due to the high accessibility, but the restrictions imposed by the authorities during the pandemic have made it difficult for the population to access. In Cismigiu Park, the posts were fewer between January and July, because those who attended the park were mostly students, who since March have conducted their courses online and have returned to the province.

**Figure 3:**
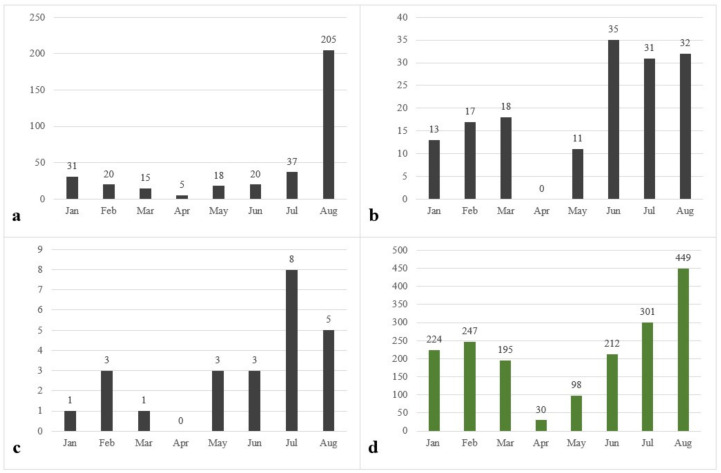
The number of posts per month for parks Cismigiu (**a**), Tineretului (**b**), Pacii (**c**) and the total number of parks (**d**).

Analyzing the results obtained from postings on social media, there is a trend of increasing the number of visitors to parks since June (Figure 3). This increase is attributed to the relaxation of the restrictions in the pandemic, but also the fact that people want to spend more time outdoors and avoid closed and crowded spaces. In August there was an increase in posts due to the warming of the weather, most of them were made regarding Cismigiu Park (210) and Tineretului Park (32 posts). The graphs (Figure 3) showing the average number of monthly recordings on Google and Instagram show the changes in the frequency of visits in parks during the COVID-19 pandemic lockdown.

The large number of posts on social media in August associated with Cismigiu Park is due to the outdoor performance of the “Bucharest of Caragiale” Theatre Festival. The festival ran over 10 weekends from 18 July to 20 September. In the case of Tineretului Park, the situation after the lockdown seems to be similar to Cismigiu Park, so the high number of posts in June, July and August reflects the organization of outdoor events. Most events in the Tineretului Park are associated with a specially designed area for children’s activities.

Following the analysis of the language of social media posts (Instagram) and reviews granted on Google, it emerged that a total of 346 posts were made on Instagram in Romanian and 329 in English, values related to the eight parks analyzed. On Google, people granted a total of 798 reviews in Romanian and 189 in English.

The results obtained for each park analyzed reflect the following aspects: most posts on Instagram in Romanian referred to Cismigiu Park, at the opposite pole with the fewest posts is Circului Park. Regarding the reviews granted on Google in English for each park, it emerged that Izvor Park has obtained 112 reviews, and no English-language posts have been identified for the Tineretului and Pacii parks. We found the average reviews of parks in the months of lockdown due to the COVID-19 pandemic to be slightly higher than the ones from regular months before it (Table 4).

Of the posts on Instagram, it emerged that most are made from personal accounts, amounting to 624. Comparing the type of account related to each park, it follows that those 251 posts from personal accounts were made about Cismigiu Park and only 19 about Circului Park. In addition to posts on personal accounts, there were posts made by various pages, resulting in a total of 64 posts. It was noted that Circului Park was the only one about which there were no posts of the pages on Instagram.

In general, social media posts come in association with check-in items to reveal a location where the person in question is located. Based on the results obtained by analyzing the posts on Instagram, we identified that a total of 640 posts that reflect photos or only a description was related to the location by using check-in. Of these, most were identified for Cismigiu Park (278), the fewest being associated with Circului Park (13). In addition to the use of check-in, the analysis of the results revealed that 27 of the posts had tag elements, met only in the case of Circului Park and Drumul Taberei. A particular case is Tei Park, where it was observed that 21 posts were accompanied only by the text of the location.

Analysis of the data obtained revealed that park visitors mentioned in their social media posts, pandemic and COVID-19 virus issues. On the Instagram platform, a total of nine mentions of the pandemic were obtained. Additionally, in the reviews on the “search engine”, Google, a total of six mentions of the virus were obtained. Thus, by reporting the total number of posts on social media, it emerged that most mentions of the pandemic were made in Cișmigiu Park (seven mentions). At the same time, these mentions were made in May and April—a period with drastic restrictions and lockdown. It is worth mentioning that the population was conditioned to stay in isolation, so people spent very little time in parks. The most encountered words in those comments included “pandemic”, “quarantine” and “mask”, others with lower frequency being: “distancing”, “lockdown”, “stay safe”, “freedom” or “Covid”.

**Figure 4:**
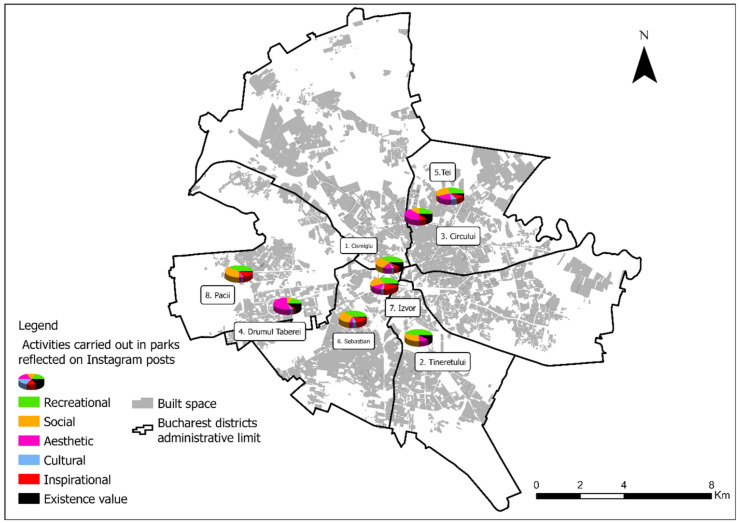
The main activities carried out in selected parks as reflected on Instagram posts (source: own elaboration).

The main activities in parks (Figure 4, Table 5) as reflected by Instagram posts are recreational activities (*n* = 506) with the higher number in the case of Cismigiu and Izvor Parks, followed by social activities (*n* = 369). High proportions are also represented by the inspirational values present in parks (*n* = 188) with lower proportions given to existence values (*n* = 89) and cultural aspects (*n* = 68).

The pictures posted on Instagram mainly depict personal activities (*n* = 333), selfies (*n* = 260) or general landscape of the park (*n* = 181). With lower frequency are present pictures of building or architectural monuments (*n* = 49), animals (*n* = 43), and plants (*n* = 42).

Following the reviews given by citizens on Google, we also looked at the aspects mentioned by them. It has emerged that people have referred in reviews mainly to the aesthetics of the parks, using words such as wide alleys, cleanliness, and greenery. They also referred to the effects that the park has on people by producing shade, air filtration, rest, and relaxation and the positive role of blue areas in parks [51]. These issues have been mentioned in all the parks we analyzed, which shows that green spaces play an important role in our daily lives. Circului Park has had numerous reviews related to the presence of water lilies on the lake in the park, but also on its naturalness and wildness, given its positioning in the center of residential urban fabric. The most used words mentioned in the reviews were beautiful, landscape, greenery, clean, quiet, and pleasant.

On the other hand, in Cismigiu Park, people noticed that at the time it was in a precarious state, had damaged urban furniture, and the alleys were dirty because of the birds that frequent quite a lot the lake area in the center of the park. The words most used in this context in all the parks analyzed were crowded, dirty, small, mosquitoes, degraded, and unkempt. The emergence of the pandemic and the establishment of a state of emergency in Bucharest in March, May and April affected the administration and care of parks massively through periods of inactivity, thus they lost their original beauty and charm.

## 4. Discussion

The peak period of the COVID-19 pandemic has strongly affected the recreation and leisure activities that people performed almost daily in the parks of Bucharest. These changes in the activities of the population are especially reflected in the evolution of posts on social media networks (Table 3 and Table 4).

This study describes a complex model of the frequency of visiting parks, observed during the COVID-19 pandemic in Bucharest. We started from a base level in January and February where there is a reduced frequency of visits in green areas, due mainly to the weather and the shorter daytime as explained in other studies [16]. Since the end of March, a state of emergency has been installed in Romania, leading to a decline in the frequency of visiting parks. Unlike Romania, a study conducted in Japan shows that the frequency of people who visited the parks during the pandemic has increased, which highlights that even during these periods, green areas are an important place where relaxation activities can take place, but also contribute to improving health [52]. A suggestion to consider would be adjusting the measures of the pandemic period, so that general population recommendations would still allow access to parks in Bucharest, of course with clearly defined protection measures. During the period of lockdowns, it is still important to provide the urban population with alternatives of enjoying the outdoors. For all of the studied parks, we observed a trend regarding the evolution of monthly posts, consisting of a sudden decrease in the number of posts from March to April, with little to no posts during this month for some parks (e.g., Tineretului and Pacii parks), continued by a steady increase in the number of posts from May onwards (Figure 3). A study on the impacts of the COVID-19 pandemic on urban park visitation in 48 countries around the world showed similar results, with a more than 80% drop in park visitor numbers for the months of March and April for most countries, followed by an increase in May [22].

This trend is explained by the response policies of the Romanian government in the face of the COVID-19 outbreak: stay-at-home restrictions, restrictions for social gatherings and cancellation of public events, as well as restrictions on internal movement. The increase in May is a consequence of the initial control of the outbreak by the authorities, which meant the relaxation of control measures. With the arrival of the warm season, restrictive measures against COVID-19 relaxed and the population began to frequent the parks in particular to carry out relaxation activities, sports, or even short walks. It can be noticed that the number of visits to the park has a pronounced ascending since June, which is also reflected in the number of social media posts.

For five out of the eight studied parks, the average review received on Google Maps improved in May compared to previous months, with Tineretului, Dr. Taberei, Tei and Sebastian parks receiving an average of 5 stars while Izvor had an average review of 4.55 stars. The surge in positive reviews for this month can be explained by the lifting of lockdown restrictions, and people finally being able to leave their homes for less urgent reasons, such as recreation and tourism.

Being reunited with urban green spaces after a prolonged period of time could have positively affected the way people perceived parks, and thus led to rating the parks more positively, as studies show that disconnection with parks during quarantine periods can negatively impact wellbeing, while having access to parks and green spaces can prevent this effect [29]. Following the month of May, the general trend for all of the parks was a decrease in the monthly average review, as people became accustomed to visiting parks.

Compared to mainstream social media platforms such as Facebook and Twitter, which provide the user with a more diverse range of posting options, Instagram and Google Maps pose limitations for the user. A study that monitored the mentions of the coronavirus on Facebook, Twitter and Instagram in Poland between February and March found that the coronavirus discourse on social media in Poland is done at a large scale, with almost 1.5 million mentions of “COVID-19” and “SARS-CoV-2” phrases recorded in the span of a month [53].

The lack of mentions related to COVID-19 in the parks’ reviews may suggest that the available public information concerning the virus was relatively saturated and the visitors did not feel the need to request or share additional information on this subject, similar to what Zhao et al. [54] observed in their study on the Chinese public’s attention to the COVID-19 epidemic on social media.

Although this study was conducted during the COVID-19 pandemic, few words related to the pandemic and the alert state in Romania were identified in the analysis of reviews found on Google. During this period, the parks became an important refuge for the inhabitants of Bucharest, and they focused in particular on the aspect of the parks, and most importantly on the benefits that the green areas bring. The post contains words such as “pandemic”, “quarantine”, and “mask”, but the word “COVID” is almost absent as it probably has a too strong emotional impact.

During the state of emergency imposed by the pandemic, society realized that green areas, especially parks, are of major importance in terms of carrying out physical and recreational activities. Most of those who frequented the parks in the pandemic chose to have recreation and relaxation activities, but there are also many people who felt the need to interact verbally or visually with other visitors. This type of interaction helps to reduce the feeling of loneliness and banishes depressive states, which is why in general parks offer safe places of social interaction for residents and help to improve mental as well as physical health. According to previous studies [3], it is important to implement more and more small private green spaces (e.g., pocket parks, residential green gardens). They would help to carry out recreational activities in a smaller area, around known people, which would increase the safety of citizens, and would prevent the panic effect, caused by the large number of visitors, as is the case with metropolitan or municipal parks as well as increased urban resilience in such events [55].

Such an analysis can represent a useful instrument for local administrations in determining both the flow of visitors but also their perceptions [56], towards the endowments, landscape and most important management of urban parks [57]. Larger volumes of data are needed for decision-making and the use of social media can prove an interesting alternative replacing classical public consultations or survey. We acknowledge that our study presents only a small sample of the situation of urban green spaces (in the case of Bucharest) and further studies should look at in-depth analysis for how different categories of urban green spaces are used.

Our results are in line with the conclusions of other studies. Previous researchers found that urban green infrastructures can play an important role in counteracting the negative impacts of COVID-19 [58]. Future planning strategies of cities will have to consider the importance of allowing access to urban green even in such periods with lockdowns enforced [59], even if we are talking about innovative forms of urban green spaces or more formalized urban parks [60].

The limitations of studies such as ours come from two main directions: data collection/analysis, and the potential to generalize and explain results. Regarding data collection we also had Twitter and Facebook as social media platforms in the initial research design, but we could not precisely locate tweets to specific parks and had great differences of gathered data between the parks in the case of Facebook (with two of them over-presented compared to the others who had a wide range of tags used for it), so in the end we decided to eliminate them from the study. For data analysis, we decided against using text-mining tools, as we considered them to be more suited for large bodies of text, such as when analyzing documents, policies or articles [Nita et al.], and as Instagram posts and Google reviews are usually reduced in size, we consider that direct coding of it suits our study better. In addition, with our current data more in-depth statistical analysis could have been realized (such as ANOVA), but we started from the assumption that we will have no significant differences between parks and we will have enough information to use words clouds as relevant for an overview of park usage during the pandemic period.

Future research direction should mainly strengthen the limits of the data collection and analysis and include more statistically and logically advanced studies. Furthermore, methods of including the natural occurring variance of park visits due to other factors (such as seasonality, weather, organized events) should be included in order to improve the comparability of the analysis of the pandemic period with that of a normal year.

## 5. Conclusions

In our study we made an assessment on how urban parks in Bucharest, Romania, were used during the COVID-19 pandemic, using data extracted from publicly available social media platforms (Instagram and Google Reviews). We analyzed the online presence of inhabitants in urban parks, evaluated their opinions as expressed in the text sections of the posts and analyzed the connection with the lockdown and restrictions imposed by the pandemic period.

We found the online presence of visitors in urban parks to be highly connected with the restrictions of the COVID-19 pandemic, but the main typologies of activities carried in the urban parks have not changed, with a strong preference for posts depicting personal activities or selfies. The reviews people gave to the park evidenced both the positive and negative features of the parks.

Further research should look upon improving the data collection procedure and including factors to differentiate park use during the pandemic from those naturally occurring variations due to external factors. Our results can be of high significance to public authorities in defining and applying their future policies related to urban green spaces. Specifically, they should consider allowing public access to parks even during pandemic periods, due to the high number of social, ecological, and economic benefits they provide to the population.

## Figures and Tables

**Table 1:** Main characteristics of the analyzed parks (visitors and visit lenght after [44]).

Park Name	Average Nr. of Visitors/Weekend Day	Average Visit Length (min)	Park Category	Accessibility of Visitors	Park Size (ha)
Cismigiu	5100	129	Metropolitan	Assured by public transport network	14.6
Tineretului	7800	113	Metropolitan	Assured by public transport network	94
Circului	3500	95	Municipal	Monopolistic position at the neighborhood level, but reduced accessibility to the rest of the city	17.2
Drumul Taberei	2200	106	Municipal	Monopolistic position at the neighborhood level, but reduced accessibility to the rest of the city	37
Tei	1200	90	District	Low	8.5
Sebastian	1100	89	District	Low	2.3
Izvor	820	97		Located near the inter-modal hubs, shopping centers, etc.	
Pacii	1100	94		Located near the inter-modal hubs, shopping centers, etc.	

**Table 2:** The number of recordings for the analyzed parks on Instagram (IG) and Google (G).

Park	January		February		March		April		May		June		July		August		Total	
	IG	G	IG	G	IG	G	IG	G	IG	G	IG	G	IG	G	IG	G	IG	G
Cismigiu	22	9	16	10	15	10	4	1	13	5	6	14	10	27	183	22	269	98
Tineretului	0	13	0	17	0	18	0	0	9	2	17	18	15	16	16	16	57	100
Circului	1	27	3	21	1	19	2	1	4	8	5	24	4	24	0	21	20	145
Dr. Taberei	0	9	1	11	2	21	3	9	7	2	2	10	5	23	3	15	23	100
Tei	3	24	2	37	3	15	0	1	3	2	11	7	26	21	32	14	80	121
Sebastian	15	43	8	25	4	15	2	0	7	3	17	7	23	21	9	17	85	131
Izvor	11	46	11	82	16	55	3	4	21	9	20	20	18	29	20	45	120	290
Pacii	0	1	3	0	1	0	0	0	3	0	3	0	8	0	4	1	22	2

**Table 3:** The number of average monthly reviews on Google.

Park Name	Average Monthly Reviews							
	January	February	March	April	May	June	July	August
Cismigiu	1.1	1.2	1.2	0.1	0.6	1.7	3.3	2.7
Tineretului	1.6	2.1	2.2	0	0.2	2.2	2	2
Circului	3.3	2.6	2.3	0.1	1	3	3	2.6
Dr. Taberei	1.1	1.3	2.6	1.1	0.2	1.2	2.8	1.8
Tei	3	4.6	1.8	0.1	0.2	0.8	2.6	1.7
Sebastian	5.3	3.1	1.8	0	0.3	0.8	2.6	2.1
Izvor	5.7	10.2	6.8	0.5	1.1	2.5	3.6	5.6
Pacii	0.1	0	0	0	0	0	0	0.1

**Table 4:** The average review by month for selected parks.

Park Name	Average Review by Month							
	January	February	March	April	May	June	July	August
Cismigiu	3.5	3.9	4.4	4	3.4	3.5	4.1	3.0
Tineretului	4.6	4.1	4.4	NA	5	4.3	4.0	3.8
Circului	4.4	4.5	4.4	5	4.3	4.5	4.7	4.4
Dr. Taberei	3.7	4.0	4.4	4.7	5	4.6	4.4	4.2
Tei	4.2	3.9	4.4	3	5	3.4	4.4	4.4
Sebastian	3.9	4.2	3.6	NA	5	3.5	4.4	4.2
Izvor	4.1	4.1	4.2	4.5	4.55	4.6	4.1	4.1
Pacii	5	NA	NA	NA	NA	NA	NA	4

**Table 5:** The main activities carried out in selected parks as reflected on Instagram posts.

	Recreational	Social	Aesthetic	Cultural	Inspirational	Existence Value
Cismigiu	39.5	25.2	14.0	3.3	7.4	10.5
Tineretului	47.9	28.2	14.1	0.0	0.0	9.9
Circului	22.6	16.1	38.7	0.0	9.7	12.9
Dr. Taberei	16.1	9.7	54.8	3.2	3.2	12.9
Tei	29.1	26.4	21.1	8.7	10.2	4.5
Sebastian	38.1	31.2	6.0	5.5	17.0	2.3
Izvor	32.0	22.3	14.6	4.9	22.9	3.4
Pacii	40.7	35.2	3.7	1.9	16.7	1.9

## Data Availability

Not applicable.
